# Changes of Taste, Smell and Eating Behavior in Patients Undergoing Bariatric Surgery: Associations with PROP Phenotypes and Polymorphisms in the Odorant-Binding Protein OBPIIa and CD36 Receptor Genes

**DOI:** 10.3390/nu13010250

**Published:** 2021-01-16

**Authors:** Melania Melis, Stefano Pintus, Mariano Mastinu, Giovanni Fantola, Roberto Moroni, Marta Yanina Pepino, Iole Tomassini Barbarossa

**Affiliations:** 1 Department of Biomedical Sciences, University of Cagliari, 09042 Monserrato, Italy; mariano.mastinu@unica.it (M.M.); tomassin@unica.it (I.T.B.); 2 Obesity Surgical Unit ARNAS G. Brotzu, 09121 Cagliari, Italy; stepintuss@gmail.com (S.P.); nannifantola@hotmail.it (G.F.); roberto.moroni@aob.it (R.M.); 3 Department of Food Science and Human Nutrition, University of Illinois, Urbana Champaign, Urbana, IL 61801, USA; ypepino@illinois.edu

**Keywords:** taste, smell, eating behavior cognitive control, bariatric surgery, gene effects

## Abstract

Bariatric surgery is the most effective long-term treatment for severe obesity and related comorbidities. Although patients who underwent bariatric surgery report changes of taste and smell perception, results from sensory studies are discrepant and limited. Here, we assessed taste and smell functions in 51 patients before, one month, and six months after undergoing bariatric surgery. We used taste strip tests to assess gustatory function (including sweetness, saltiness, sourness, umaminess, bitterness and oleic acid, a fatty stimulus), the “Sniffin’ Sticks” test to assess olfactory identification and the 3-Factor Eating Questionnaire to assess eating behavior. We also explored associations between these phenotypes and flavor-related genes. Results showed an overall improvement in taste function (including increased sensitivity to oleic acid and the bitterness of 6-n-propylthiouracil (PROP)) and in olfactory function (which could be related to the increase in PROP and oleic acid sensitivity), an increase in cognitive restraint, and a decrease in disinhibition and hunger after bariatric surgery. These findings indicate that bariatric surgery can have a positive impact on olfactory and gustatory functions and eating behavior (with an important role of genetic factors, such PROP tasting), which in turn might contribute to the success of the intervention.

## 1. Introduction

It is known that obesity is a complex and multifactorial disease that originates from a combination of social, cultural, environmental, genetic, behavioral, metabolic and endocrinological factors [1]. Today, obesity and overweight affect over a third of the world’s population [2,3]. Furthermore, overweight and obesity are considered risk factors for serious health morbidities such as hypertension, type 2 diabetes, hypercholesterolemia, cardiovascular disease, and some types of cancers [4]. Obesity is correlated with eating habits characterized by stronger preferences for energy dense foods, such as fats and sweets [5,6,7,8], which leads to greater consumption of these kind of foods [9,10,11]. These unbalanced eating habits could find explanation in a reduction in the gustatory and olfactory sensitivities or blunted brain reward activation in response to palatable food, which has been observed in people with overweight and obesity [12,13,14,15,16,17], as well as in preclinical models of obesity [18]. Reductions in the taste sensitivity for sweet [19], umami [20], bitter and sour [11], and fatty acids [21], and impaired olfactory performance have been observed in people with obesity [11,22,23,24,25].

Variations of taste and olfactory sensitivities can be due to sundry factors (e.g., genetics, environment and age) which can thus constitute risk factors for the onset of overweight and obesity. Among them, the genetic ability to taste the bitterness of the 6-n-propylthiouracil (PROP) has been proposed as an oral marker of general taste perception, food preferences and dietary behavior with subsequent impacts on body composition and health [26,27,28,29,30,31,32,33,34,35,36,37,38,39,40,41,42,43,44,45,46,47,48,49,50,51]. Individual variability of the PROP taste sensitivity is mostly due to the allelic diversity of the PROP-binding bitter receptor gene, *TAS2R38* [52,53], which gives rise to two common haplotypes: the taster variant (PAV) and the non-taster one (AVI) [35,52], as well as to rare haplotypes with intermediate sensitivities [54,55]. Genetic factors are also involved in the variability of fat sensitivity and dietary preferences. Among them, the *rs1761667* (G/A) polymorphism in the gene coding for the CD36 receptor [31,56,57,58,59] is primarily responsible for the detection of long chain fatty acids [57,60,61]. The substitution of allele A for G has been associated with a decrease in sensitivity to fatty acids [31,57,62,63,64], expression of CD36 protein [65,66] and metabolism [67]. Interestingly, the fatty acid flavor has been associated with the polymorphism *rs2590498* (A/G) of the gene coding for the odorant-binding protein OBPIIa [68,69], with participants with the A allele being generally more sensitive than those with the G allele. This polymorphism has also been associated with bitter taste perception of PROP [68] and with olfactory performance in healthy subjects or women with Parkinson’s disease [70,71].

Bariatric surgeries, such as sleeve gastrectomy (SG) and Roux-en-Y gastric bypass (RYGB), are the most effective long-term treatments for severe obesity and of its related comorbidities [72,73,74,75]. SG is a bariatric technique consisting of subtotal vertical gastrectomy with preservation of the pylorus, including longitudinal resection of fundus, corpus and antrum, to create a tubular duct along the lesser curvature. The resection comprises approximately 80% of the stomach and the remnant gastric has a capacity of < 100 mL. SG is considered to be an easier technique compared to other procedures such as RYGB which require multiple anastomoses [76]. In RYGB, the stomach is also reduced to a small pouch that is connected to the small intestine, bypassing the duodenum and the proximal part of the jejunum.

Patients with obesity who undergo bariatric surgery report changes of taste, smell, appetite, and food preferences [77,78,79]. Specifically, after undergoing surgery, patients report a preference for low calorie foods [80], reduced interest for sweet and high-fat food [12,81,82,83,84,85,86] and a specific aversion for sweet, high calorie foods and meats [78,87,88]. Although patients’ self-reports that surgery changed their taste perception are ubiquitous, findings from sensory studies that evaluated taste and smell perception in bariatric patients are inconsistent and, to the best of our knowledge, there is no published data on how these surgeries might affect fat sensitivity. For example, while some authors found that patients became more sensitive to bitter and sour tastes (but not sweet or salty) following surgery [89], others found increased sensitivity to sweet [87,90], but not bitter, taste [87]. Yet, others found no changes in taste sensitivity or perceived intensity of sweetness, saltiness, or savoriness [85,91]. Interestingly, using taste strip tests on patients who mostly underwent SG, Holinski et al. found increased taste identification after 6 months of surgery [92]. A result that was replicated by Altun and collaborators in patients who were evaluated before and after 3 months of SG [93]. In comparison to surgery-related changes in taste function, fewer studies have assessed olfactory function, albeit there is discrepancy on study findings for smell as well. Using sniffin sticks, Holinskly and collaborators found that olfactory function improved after 6 months postsurgery to a level that was close to that of normal-weight subjects [92], and Hanci and colleagues found increased sensitivity, discrimination and identification parameters of smell function [94]. However, Jurowich and colleagues found only an decrease in the threshold [95] and Enck and collaborators found no changes in either threshold, discrimination or identification [96]. Using scratch and sniff tests, Zerrweck and collaborators [97] found increases in odor identification 6 months post RYGB surgery, but Richardson and colleagues found no changes in smell identification [98]. Although these findings on the taste and smell changes following bariatric surgery are unclear and limited, they suggest that variations in gustatory and olfactory functions could underly changes in eating choices [83], the reduction in the consumption of high-calorie foods and, therefore, contribute to the success of the intervention.

The main goal of this study was to determine the effects of bariatric surgery on several well-established sensory phenotypes, including PROP tasting, olfactory and gustatory perception of the five basic taste qualities (sweet, salty, sour, bitter, umami) and, for the first time, we evaluated its impact on oral fat sensitivity. The effects of bariatric surgery on three important dimensions of eating behavior (dietary restraint, disinhibition and perceived hunger), which could confound relationships between taste and olfactory sensitivities/food choices/metabolism [99,100,101,102,103,104,105,106,107], and can be profoundly affected by surgery [108,109,110,111,112], as well as parameters defining body composition were also determined.

Since individual differences in sensory responses are partially controlled by genetic factors, as a secondary aim we also explored whether the effects of bariatric surgery interacted with gene polymorphisms which have been previously associated with taste and olfactory sensitivity. Specifically, (1) *TAS2R38* polymorphisms, (2) the *r1761667* (G/A) polymorphism of the *CD36* gene and (3) the *rs2590498* (A/G) polymorphism of the *OBPIIa* gene.

## 2. Materials and Methods

### 2.1. Participants

Sixty-eight Caucasian participants who were scheduled to undergo bariatric surgery were initially recruited at the Bariatric surgery Center, G. Brotzu Hospital (Cagliari, Italy) for the study. However, fifty-one of them (15 men and 36 women; age 43.5 ± 1.5 y; body mass index (BMI): 43.0 ± 0.8 kg/m^2^, range 33.1–59.2 kg/m^2^) who were scheduled to undergo either sleeve gastrectomy (SG) (*n* = 21), Roux-en-Y gastric bypass (RYGB) (*n* = 26) or mini gastric bypass (*n* = 4) participated in this study, while seventeen subjects left the study after surgery. The initial recruitment of subjects involved reviewing medical records and in-person interviews conducted by a multidisciplinary team with surgical, nutritional, and psychological expertise. We excluded potential participants who had a diagnosis of a major disease (e.g., diabetes or kidney disease), were pregnant or breastfeeding, had food allergies, were on medications that could alter taste or affect lipid metabolism, or had undergone a previous gastrointestinal surgery.

### 2.2. Experimental Procedure

Each participant was tested in three separate visits: before bariatric surgery (T0) and one month (T1) and six months (T2) after surgery. The same experimental procedures were completed in all three visits. At 9.00 am, participants arrived at test room after they fasted for at least 12 h at home. All sensory studies were conducted in a room with good temperature control (23–24 °C; 40–50% relative humidity). Parameters defining body composition were determined for each participant as described below. Participants were assessed for cognitive control of eating behaviors by the 3-Factor Eating Questionnaire (TFEQ) [113] which estimates three aspects of eating behavior: dietary restraint, disinhibition and perceived hunger. All completed a battery of sensory tests to assess their PROP taster status, their taste sensitivity for the six taste qualities (sweet, salty, sour, bitter, umami and fat) and olfactory function.

In the first visit, a sample of blood (4 mL) was collected, promptly centrifuged and stored at −80 °C until the molecular analyses described below were completed.

### 2.3. Anthropometric Determinations

Body weight (BW, kg) and height (m) were measured (Wunder^®^, Trezzo sull’Adda, Italy) in each participant in order to calculate the body mass index (BMI, kg/m^2^). Neck, waist and hip circumferences were measured and the waist-to-hip ratio (WHR) was calculated. The percentage of total weight loss (% TWL) and percentage of excess weight loss (% EWL), which is defined as percentage of lost weight that is in excess with respect to the ideal body weight (IBW), were determined at T1 and T2. The IBW was calculated considering an ideal BMI of 25 kg/m^2^.

Bioelectrical impedance analysis (BIA) obtained by Bodygram Plus^®^ (Akern^®^, Pontassieve, Italy), was used to estimate body composition and total body water (TBW). The analyzer can be used for subjects with weights of up to 200 kg. The measuring of body composition was performed by using a constant current source at a frequency of 50 kHz and 8 mÅ, which was applied to each participant by using two copies of positioned electrodes—the first copy in the back of the hand and in the instep, and the second one in the wrist and in the ankle. BIA provides an estimate of TBW (in liters with an approximation of 0.1 L) by which it is possible to estimate fat-free mass (FFM) and fat mass (FM) (both measured in kilograms with an approximation of 0.1 kg). The percentage of hydration (%TBW) was calculated as the ratio between TBW and FFM.

### 2.4. PROP Taster Status Classification

Participants were classified for their PROP taster status by using the impregnated paper screening test [114], a validated psychophysical approach that has been in used in previous studies [115,116]. In brief, two paper disks—one impregnated with sodium chloride, NaCl (1.0 mM) and the other one with PROP solution (50 mM)—were sequentially placed on the tip of the subject’s tongue for 30 s and subjects rated their perceived taste intensity by using the Labeled Magnitude Scale (LMS) [117]. Participants were instructed to use the LMS scale to evaluate intensity of PROP bitterness relative to the strongest imaginable oral stimulus ever perceived. The LMS is a semilogarithmic 100 mm scale in which 7 verbal descriptors (barely detectable, weak, moderate, strong, very strong and strongest imaginable) are organized along the length of the scale [117]. Participants who gave ratings above 67 mm on the LMS for the PROP disk were classified as PROP super-tasters; those who gave ratings below 15 mm were classified as PROP non-tasters, and those who gave intermediate ratings were classified as PROP medium tasters [114]. For participants who gave a borderline rating for the PROP disk (i.e., unclear classification), the PROP taster group assignment was decided by comparing their PROP ratings relative to their NaCl ratings, since the taste intensity to NaCl does not change with PROP taster status in this procedure [118].

### 2.5. Taste Sensitivity Measurements

Taste sensitivity to the four basic qualities (sweet, sour, salty, bitter) was evaluated by using the Taste Strip Test (TST, Burghart Company, Wedel, Germany) [119,120]. Sixteen filter paper strips impregnated with four concentrations of stimuli representative of four basic taste qualities (i.e., sweet: 0.05, 0.1, 0.2, and 0.4 g/mL of sucrose; sour: 0.05, 0.09, 0.165, and 0.3 g/mL of citric acid; salty: 0.016, 0.04, 0.1, and 0.25 g/mL of NaCl; bitter: 0.0004, 0.0009, 0.0024, and 0.006 g/mL of quinine hydrochloride) were presented to participants in a pseudorandomized manner (although concentrations within each solution type were presented in increasing concentrations). Participants placed each paper strip on the tongue and identified, from a list of four descriptors (sweet, sour, salty, and bitter), the taste quality they perceived. Each correct identification was rated as 1. Therefore, the maximum score for each taste quality was four and that for the whole TST was 16. Each subject was also tested for her/his sensitivity for the umami taste. Four filter papers impregnated with 10 µL of monosodium glutamate (MSG) solutions (0.0017, 0.0085, 0.0170 and 0.0338 g/mL) were used. Each correct identification was rated as 1 and the maximum score was 4. The interstimulus interval was set at 60 s and before each stimulation subjects rinsed their mouths with spring water.

### 2.6. Oleic Acid DetectionThreshold Assessments

The detection thresholds for oleic acid were evaluated in each participant, in the absence of a nose clip, by a variation of the staircase approach implemented in a 3-Alternative Forced Choice (3-AFC) test according to Melis et al. [31]. Participants were presented with 3 paper filter disks: 2 impregnated with 10 µL of mineral oil (control) and 1 with the amount of oleic acid under evaluation. Patients placed the paper disk on the center of their tongue, kept it in the mouth for 10 s and then spat it out. Oleic acid samples were tested in ascending order, from the lowest concentration (0.0015 µL) to the highest (pure), until subjects correctly identified the odd sample in two consecutive trials. The oleic acid concentration was increased after a single incorrect response and decreased after 2 correct responses in a row. A reversal was considered a point where the concentration sequence changed direction. The threshold concentration was calculated as the mean value of the 4 reversals. Participants rinsed their mouths after each triad. The time between triads was 2 min.

### 2.7. Olfactory Function Assessments

Olfactory function assessments of each participant were evaluated by using the odor identification part of the “Sniffin’ Sticks” test (SSET) (Burghart, Wedel, Germany) [121]. Assessments were based on the participant’s ability to identify 16 different odors presented by using felt-tip pens [122]. The same procedure was repeated in each participant for the 16 odors. After removing the cap, the pen tip releasing the odor was positioned 2 cm in front of the participant’s nostrils for 2 s, and then the pen was capped. Participants had to identify, for each pen, the odor they smelled using a multiple-choice task from a list of four descriptors, which were proposed by a card showing their pictures and names. According to the forced choice option, participants had to choose a descriptor, even if they were unsure that they smell anything. The interval between odor presentations was 20–30 s. The subject identification score (IdS) corresponds to the number of correct identifications and ranged from 0 to16. The classification of each participant as normosmic or non-normosmic was decided based on previous scores that take into account their age and gender [121]. The cut off values for normosmia for those in the age group 36–55 y were: 11 for men and 12 for women. For those older than 55 y, the values were nine for both sexes.

### 2.8. Molecular Analysis

DNA was extracted from blood samples by using the QIAamp^®^ DNA Mini Kit (QIAGEN S.r.l., Milan, Italy) according to the manufacturer’s instructions. Its concentration was assessed by measurements at an optical density of 260 nm with an Agilent Cary 60 UV–Vis Spectrophotometer (Agilent, Palo Alto, CA, USA).

Subjects were genotyped for the following single nucleotide polymorphisms (SNPs): the three SNPs, *rs713598*, *rs1726866*, and *rs10246939*, of *TAS2R38*, which result in three amino acid substitutions (Pro49Ala, Ala262Val, and Val296Ile) and give rise to two major haplotypes, PAV (the dominant taster variant) and AVI (the nontaster recessive one) and three rare haplotypes (AAI, AAV, and PVI); the *rs1761667* (G/A) SNP of *CD36*, the *rs2590498* (A/G) SNP of the gene coding for the olfactory binding protein OBPIIa.

To genotype the SNPs of *TAS2R38* and *CD36,* molecular analyses were performed by using TaqMan SNP Genotyping Assay (C_8876467_10 assay for the *rs713598*; C_9506827_10 assay for the *rs1726866* and C_9506826_10 assay for the *rs10246939;* C_8314999_10 assay for the *rs1761667*) according to the manufacturer’s specifications (Applied Biosystems by Life Technologies Milano Italia, Europe BV). For the *rs2590498* (A/G) SNP of *OBPIIa* gene, a custom TaqMan^®^ SNP Genotyping Assay was used according to our previous work [68,69]. The fluorescence of plates was read (60 °C for 1 min) in the sequence detector system, and the results were analyzed by allelic discrimination by the sequence detector software (Applied Biosystems). Replicates and positive and negative controls were included in all reactions.

### 2.9. Statistical Analyses

Separate repeated measures ANOVAs were used to compare the differences in anthropometric parameters, PROP bitterness intensity, total taste score of the whole TST, taste score of each taste quality (sweet, sour, salty, bitter and umami), oleic acid detection threshold, odor identification score (IdS) and three factors of the TFEQ [113] across time—i.e., before (T0), and at one month (T1) and six months (T2) after bariatric surgery. Data were also separately analyzed according to *CD36* and *OBPIIa* polymorphisms or PROP taster status. We ran the same data analyses including gender or type of surgery (SG vs. RYGB including mini bypass) in the model. A main effects ANOVA was used to assess the first order (noninteractive) effects of multiple categorical independent variables. When the sphericity assumption was violated, we used the Greenhouse–Geisser correction or Huynh–Feldt correction to modify the degrees of freedom. Post hoc comparisons were performed with the Fisher’s least significant difference (LSD) test. The effect of age as covariate in repeated measures ANCOVAs was controlled for all parameters. No significant effect of age was found. Therefore, these data are not reported. One-way ANOVA was used to compare differences in age according to type of surgery, PROP taster status in each time sampling (T0, T1 and T2), and *TAS2R38*, *CD36* or *OBPIIa* loci. The genotype distribution and haplotype frequency of the *TAS2R38* locus were tested at T0, T1 and T2 according to PROP taster status by the Fisher method (Genepop software version 4.2; http://genepop.curtin.edu.au/genepop_op3.html) [123]. Fisher’s Exact Test was used to analyze differences in the number of participants classified as PROP super-tasters, PROP medium tasters and PROP non-tasters in each sampling time. Statistical analyses were conducted using STATISTICA for Windows (version 10; StatSoft Inc., Tulsa, OK, USA). The significance level was set at *p* < 0.05.

## 3. Results

### 3.1. Participants’ Demographic, Clinical and Genetic Features

Demographic, clinical features and the genotype distribution of the gene polymorphisms determined in the obese participants are shown in Table 1. Molecular analysis at the three SNPs of the *TAS2R38* locus identified 10 subjects who were PAV homozygous, 19 who were heterozygous, and 18 who were AVI homozygous. Rare haplotypes were also found in four participants: three carried the PAV/AAV genotype and one the AAV/AVI genotype. Participants with rare haplotypes were excluded from sensory data analyses. Molecular analysis at the SNP (*rs1761667*) of the *CD36* locus identified 14 subjects who were homozygous GG, 23 who were heterozygous, and 14 who were homozygous AA. In addition, 15 subjects were homozygous AA for the SNP (*rs2590498*) of the *OBPIIa* locus, 12 were heterozygous, and 24 were homozygous GG.

No difference in the age of subjects who underwent the different types of bariatric surgery or belonging to each genotype groups were found (*p* > 0.05; one-way ANOVA; data not shown).

The effects of bariatric surgery on anthropometric parameters defining the body composition (mean values ± SEM determined before (T0), one month (T1) and six months (T2) after bariatric surgery) are shown in Supplementary Materials Table S1.

### 3.2. Bariatric Surgery-Induced Effects on PROP Tasting

**Figure 1:**
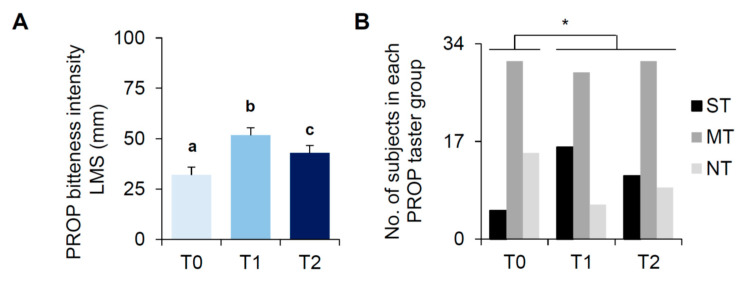
6-N-propylthiouracil (PROP) tasting before (T0), one month (T1) and six months (T2) after bariatric surgery. Means (±SEM) values of PROP bitterness intensity ratings (50 mM) (**A**) and numbers of subjects classified as super-tasters (STs), medium tasters (MTs), and non-tasters (NTs) (**B**). (*n* = 51). Different letters in (**A**) indicate significant difference (*p* ≤ 0.037, Fisher’s test least significant difference (LSD) subsequent repeated measures ANOVA). * in (**B**) indicates a significant difference (*p* = 0.0078; Fisher’s exact).

PROP tasting changes associated with bariatric surgery-induced weight loss are shown in Figure 1. The repeated measures ANOVA showed that PROP bitterness intensity ratings increased after the bariatric surgery (F_(2,100)_= 10.724; *p* = 0.00006) (Figure 1A). Post hoc comparison showed that the PROP intensity ratings determined at T1 and T2 were higher than that measured at T0 (*p* ≤ 0.016; Fisher’s test LSD), and the intensity rating determined at T2 was lower than that determined at T1 (*p* = 0.031 Fisher’s test LSD). No difference related to gender or type of bariatric surgery was found (*p* > 0.05; data not shown).

Similarly suggesting a shift towards increased PROP sensitivity after surgery, the proportion of participants who were classified as PROP super-tasters, medium tasters, and non-tasters at T0 differed to those at T1 and T2 (χ^2^ > 7.72; *p* < 0.0073; Mc Nemar test) (Figure 1B). Specifically, super-tasters increased after surgery (T0: 9.8%, T1: 31.4%, T2: 21.6%), non-tasters decreased after surgery (T0: 29.4%, T1: 11.8%, T2: 17.7%), while medium tasters did not change after surgery (T0: 60.78%, T1: 56.86%, T2: 60.78%). Medium tasters were younger than super-taster and non-tasters at T0 (*p* < 0.044). No other differences in age of subjects belonging to each PROP taster group were found (*p* > 0.05; one-way ANOVA; data not shown).

The genotype distribution and haplotype frequency for SNPs of *TAS2R38* according to PROP taster status determined at T0, T1, and T2 are shown in Table 2. PROP taster groups differed statistically on the basis of the genotype distribution and haplotype frequency at T0, T1 and T2 (genotype: χ^2^> 12.439; *p* < 0.0019; haplotype: χ^2^> 11.927; *p* < 0.00257; Fisher’s method). Post hoc comparison also showed that the nontaster group differed from the other ones at all time of assessments (genotype: χ^2^ > 8.45; *p* < 0.014; haplotype: χ^2^ > 11.188 *p* < 0.0037; Fisher’s method), while no difference between super-tasters and medium tasters was found (genotype: χ^2^ > 5.198; *p* > 0.074; haplotype: χ^2^ > 5.439; *p* < 0.065; Fisher’s method). The genotype AVI/AVI and haplotype AVI were more frequent in non-tasters (genotype: T0: 73.33%; T1: 100%; T2: 88.89%; haplotype: T0: 86.67%; T1: 100%; T2: 94.44%), while the genotype PAV/AVI was more frequent in super-tasters and medium tasters. The prediction of PROP taster groups by genotype and haplotype at *TAS2R38* varied with the time of assessment (i.e., T0, T1 and T2). Participants with a PAV haplotype were more likely to be classified as a super-taster after (T1 or T2) than before (T0) surgery (T0: 10%, T1: 41 %, T2: 36%; χ^2^ = 10.28; *p* < 0.0058; Mc Nemar test) and subjects with the AVI haplotype were more likely to be classified as a non-taster before than after surgery (T0: 47%, T1: 22% and T2: 31%; χ^2^ = 8.236; *p* < 0.016; Mc Nemar test). The statistical differences with and without inclusion in the analysis of participants with rare haplotype were the same.

### 3.3. Bariatric Surgery-Induced Effects on Scores of Taste Perception

**Figure 2:**
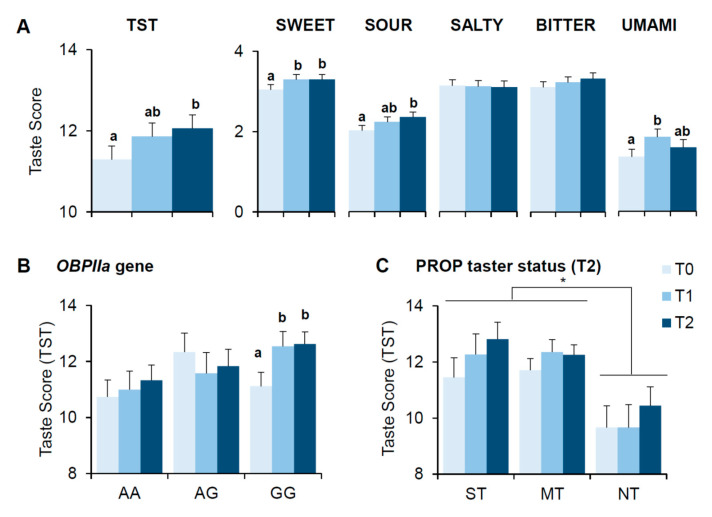
Taste perception scores determined before (T0), one month (T1) and six months (T2) after bariatric surgery. Means (±SEM) values of the total taste score of the whole Taste Strip Test (TST) and of that relative to sweet, sour, salty, bitter and umami (*n* = 51) (**A**). Data of the total taste score of the whole TST are shown according to the *rs2590498* polymorphism of *OBPIIa* gene (genotypes AA: *n* = 15; genotypes AG: *n* = 12; genotypes GG: *n* = 24) (**B**) or PROP taster status determined at T2 (super-tasters: *n* = 11; medium tasters: *n* = 31; non-tasters: *n* = 9) (**C**). Different letters indicate a significant difference (*p* ≤ 0.048, Fisher’s test LSD subsequent repeated measures ANOVA). * indicate a significant difference between values of tasters and non-tasters *(p* ≤ 0.027 Fisher’s test LSD subsequent repeated measures ANOVA).

The taste score changes associated with bariatric surgery-induced weight loss are shown in Figure 2. The mean values (±SEM) of the total taste score for the whole TST and of that relative to sweet, sour, salty, bitter and umami determined before (T0), one month (T1) and six months (T2) after bariatric surgery are shown in Figure 2A. Data of the total taste score of the whole TST are shown also according to the *rs2590498* polymorphism of *OBPIIa* gene in Figure 2B and for each PROP taster group determined at T2 in Figure 2C.

The repeated measures ANOVA showed that the total taste score varied with the time factor (T0, T1 and T2) (F_(1.84,91.85)_ = 3.509; *p* = 0.038) and post hoc comparison showed that the total taste score determined at T2 was higher than that determined at T0 (*p* = 0.0122, Fisher’s test LSD). Changes in sweet, sour and umami scores were the major contributors to the total taste score changes across time (sweet: F_(1.78,88.94)_=2.978; *p* = 0.059; sour: *F*_(2,100)_ = 3.38; *p* = 0.038; umami: F_(2,100)_=2.995; *p* = 0.054). Post hoc comparison showed that while sweet and umami scores increased already at T1 (*p* ≤ 0.045, Fisher’s test LSD), the sour score increased only at T2 (*p* = 0.0118, Fisher’s test LSD). No differences in salty and bitter scores were found (*p* > 0.05) (Figure 2A).

Repeated measures of ANOVA also showed that the changes in total taste score across time were associated with the *OBPIIa* gene polymorphism (F_(4,96)_ = 2.836; *p* = 0.0284) (Figure 2B). Specifically, the total taste score of participants who carried the GG genotype increased already at T1 (*p* < 0.0011, Fisher’s test LSD), while no differences in the total taste score across time were found in participants who carried the AA or AG genotypes (*p* > 0.05). Differently, the change of the total taste score observed with the time factor (T0, T1 and T2) did not associate with PROP taster status of participants. However, a significant main effect of the PROP taster status on the total taste score was found (F_2,148)_ = 10.762; *p* = 0.00004), such that super-tasters and medium tasters had higher scores than non-tasters (*p* ≤ 0.000074, Fisher’s test LSD) (Figure 2C). No other difference related to PROP taster status was found (*p* > 0.05).

There were no significant differences of total taste score or scores relative to each taste quality related to gender or type of bariatric surgery (*p* > 0.05; data not shown).

Details of effects of the polymorphism of *OBPIIa* gene, or PROP taster status, on the scores of sweet, sour, salty, bitter and umami taste perception determined before (T0), one month (T1) and six months (T2) after bariatric surgery are shown in Figure S1. The repeated measures ANOVA showed that the association between the total taste score changes across time with *OBPIIa* locus was mainly due to changes in sweet and sour scores (sweet: F_(3.66,87.94)_ = 3.169; *p* = 0.020; sour: F_(4,96)_ = 4.107; *p* = 0.0041).

### 3.4. Bariatric Surgery-Induced Effect on Oleic Acid Detection Thresholds

**Figure 3:**
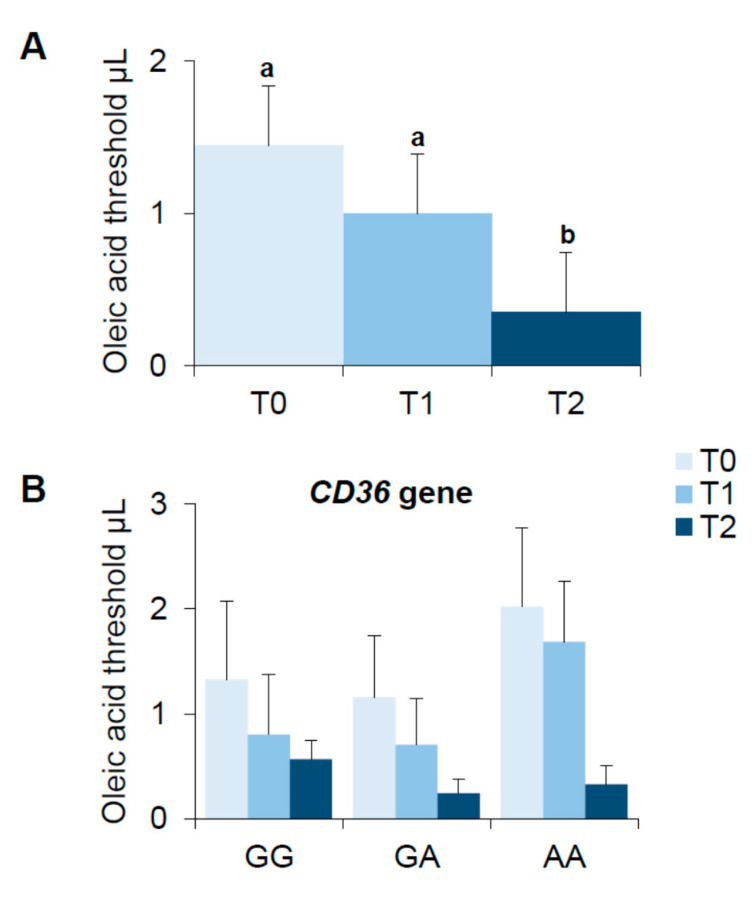
Means (±SEM) values of the oleic acid threshold (μL) determined before (T0), 1 month (T1) and 6 months (T2) after bariatric surgery (**A**). The same data are shown for each genotype of the *rs1761667* (A/G) polymorphism of *CD36* gene (genotypes GG: *n* = 14; genotypes GA: *n* = 23; genotypes AA: *n* = 14) (**B**). (*n* = 51). Different letters indicate a significant difference (*p* ≤ 0.043, Fisher’s test LSD subsequent repeated measures ANOVA).

Figure 3 shows mean values (±SEM) of the oleic acid detection threshold determined before (T0), one-month (T1) and six months (T2) after bariatric surgery (Figure 3A). The repeated measures ANOVA showed that oleic acid threshold varied with the time factor (T0, T1 and T2) (F_(1.8,90.06)_ = 6.028; *p* = 0.0047). Post hoc comparison showed that the oleic acid detection threshold determined at T2 was lower than those measured at T0 and T1 (*p* ≤ 0.043, Fisher’s test LSD). When results were analyzed according to the *rs1761667* polymorphism of *CD36*, the decrease in the oleic acid detection threshold observed after bariatric surgery did not depend on the *CD36* locus, and all genotype groups showed the same trend—i.e., oleic acid thresholds were decreased at T2 compared to T0 (Figure 3B).

No significant differences of oleic acid threshold related to gender or type of bariatric surgery were found (*p* > 0.05; data not shown).

### 3.5. Bariatric Surgery-Induced Effect on Olfactory Function

**Figure 4:**
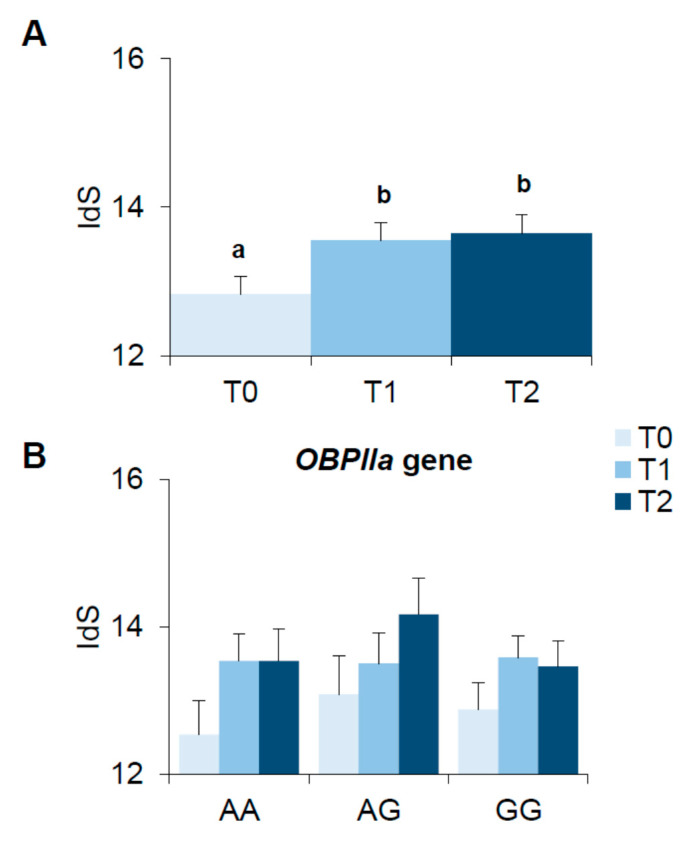
Means (±SEM) values of the odor identification score (IdS) determined before (T0), one month (T1) and six months (T2) after bariatric surgery (*n* = 51) (**A**). The same data are shown for each genotype of the *rs2590498* polymorphism of *OBPIIa* gene (genotypes AA: *n* = 15; genotypes AG: *n* = 12; genotypes GG: *n* = 24) (**B**). Different letters indicate a significant difference (*p* ≤ 0.0056, Fisher’s test LSD, subsequent repeated measures ANOVA).

The olfactory function of participants improved after bariatric surgery. Figure 4 shows mean values (±SEM) of the odor identification score (IdS) determined before (T0), one month (T1) and six months (T2) after bariatric surgery (Figure 4A). The repeated measures ANOVA showed that the IdS varied with the time factor (T0, T1 and T2) (F_(2,100)_ = 9.104; *p* = 0.00023), with higher values determined at T1 and T2 with respect to T0 (*p* ≤ 0.00084, Fisher’s test LSD). Analysis of the same data according to *OBPIIa* locus showed that the increase in IdS values observed after bariatric surgery was not associated with the *OBPIIa* locus (*p* > 0.05), and all genotype groups showed improvement of olfactory function after bariatric surgery (Figure 3B). There were no differences in IdS scores determined before or after surgery between participants who underwent the different types of bariatric surgery or related to gender (*p* > 0.05; data not shown).

### 3.6. Bariatric Surgery-Induced Effects on Scores of the 3-Factor Eating Questionnaire (TFEQ)

**Figure 5:**
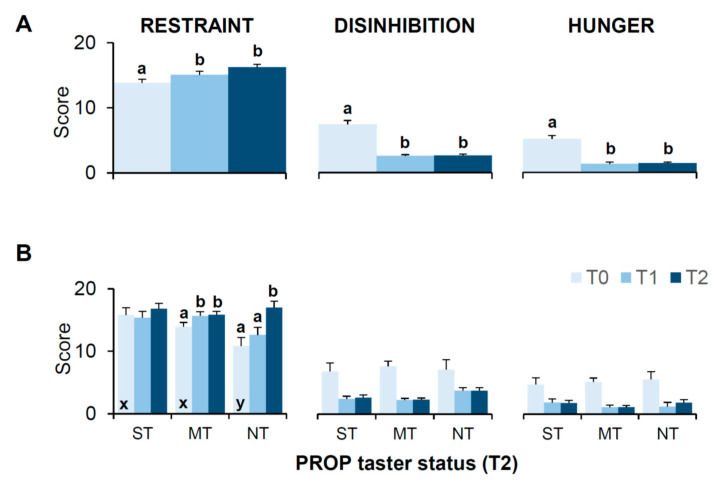
Mean (± SEM) values of the scores of the Three-Factor Eating Questionnaire (TFEQ) determined before (T0), one month (T1), and six months (T2) after bariatric surgery (**A**). The same data are shown for each PROP taster group determined at T2 (super-tasters: *n* = 11; medium tasters: *n* = 31; non-tasters: *n* = 9) (**B**). Significant differences are indicated by different letters: a and b are used to denote differences within sampling time (T0, T1 or T2), while x and y are used to denote differences with respect to the corresponding value of other groups. For all comparisons (*p* ≤ 0.027, Fisher’s test LSD subsequent repeated measures ANOVA).

Figure 5 shows mean values (±SEM) of the scores of the 3-Factor Eating Questionnaire (TFEQ) of Stunkard and Messick [113], assessed before (T0), one month (T1), and six months after surgery (T2). The same data are shown for each PROP taster group determined at T2 (Figure 5B).

The repeated measures ANOVA showed that the values of restraint, disinhibition and hunger varied with the time factor (T0, T1 and T2) (restraint: F_(2,100)_ = 7.353, *p* = 0.0011; disinhibition: F_(1.24,59.52)_ = 52.908, *p* < 0.00001; perceived hunger: F_(1.51,72.74)_ = 48.461, *p* < 0.00001) (Figure 5A). Post hoc comparison showed that, compared to score values attained at T0, values of restraint increased (*p* < 0.05; Fisher’s test LSD) and values of disinhibition and perceived hunger decreased (*p* < 0.000001; Fisher’s test LSD) at T1 and T2 (Figure 5A).

The repeated measures ANOVA showed that the restraint scores determined at the three sampling times were associated to participant’s PROP taster status (F_(3.76,86.41)_ = 2.688, *p* = 0.039). Post hoc comparison showed that compared to PROP tasters, PROP non-tasters had lower restraint scores at baseline (T0) (*p* ≤ 0.025; Fisher’s test LSD). Post hoc comparison also showed that the restraint scores of medium tasters increased already at T1 with respect to T0 (*p* ≤ 0.027, Fisher’s test LSD), while those of non-tasters increased only at T2 (*p* ≤ 0.0045, Fisher’s test LSD) (Figure 5B). No differences in super-tasters were found related to sampling time (*p* > 0.05). No effect of PROP taster status on disinhibition and hunger scores was found (*p* > 0.05).

There were no differences in the restraint or perceived hunger scores related to types of bariatric surgery or gender (*p* > 0.05; data not shown), although, overall, women had a basal level of disinhibition that was higher than that of men (F_(1.24, 58.53)_ = 4.169, *p* = 0.0037; *p* ≤ 0.0040, Fisher’s test LSD).

## 4. Discussion

The main finding of this study is that bariatric surgery is associated with increased taste and smell identification, as well as with weight loss and improvement of body composition. Interestingly, we found an overall increase in taste sensitivity to PROP bitterness and general taste sensitivity, although changes in taste score after surgery were mostly explained by increasing identification of sweet, sour and umami stimuli with no changes in salty stimuli or in identification of bitterness when tasting quinine. The present study also documented, for the first time, an increased sensitivity to fatty acids.

Interestingly, the increase in PROP sensitivity after surgery was not only evident by patients’ reports of increased bitterness intensity ratings, but also by the increased number of subjects that were classified as super-tasters at the expense of those classified as non-tasters. It may be worth mentioning that PROP contains the functional group (SC(NHR)2) which is responsible for its bitter taste [124,125,126]. This chemical moiety is also a component of naturally occurring glucosinolates that are widely present in plants, particularly of the family of Brassicaceae. PROP responsiveness is associated with bitterness of these glucosinolate-containing products [127], thus may provide a direct connection between responses to bariatric surgery and dietary preferences. The increased sensitivity to PROP bitterness that we found at T1 might be related to the very low-calorie diet in the first few months after surgery. In fact, data from preclinical models show that T2R gene expression is regulated by cholesterol-sensitive SRBP2, so that diets low in fat sensitize bitter signaling to increase sensitivity to possible plant toxins [128]. Differently, Hubert and colleagues did not find variation in the frequency of the PROP taster categories between the pre- or postsurgery groups [129], which might be due to the fact that they used a cross-sectional study design unlike the current study which used a longitudinal design. In other words, we found that subjects with the PAV variant were more likely classified as a super-tasters after as opposed to before surgery and subjects with the AVI variant were more likely classified as non-tasters before as opposed to after surgery. We also observed that many of the AVI/AVI subjects could detect PROP after surgery. Together, these results suggest that the increase in PROP bitterness sensitivity after surgery is supported by a mechanism different to that mediated by TAS2R38 receptor. Although variants in *TAS2R38* account for most of the PROP phenotype variance, other genetic and nongenetic modifiers exist and could became dominant after surgery. For example, previous research suggests that other receptors in the T2R family [130] and other non-bitter receptor genes [131,132,133] modify PROP taste ability. Previous research also suggests other modifiers, including receptor cell number and density [115,134,135], development and disease [135,136,137].

That increased taste identification scores postsurgery are in agreement with results from previous authors who also used the taste strip test to assess taste function in bariatric population [92,93]. We found that changes in sweet, sour, and umami scores were the major contributors to the overall taste score changes across time, with increases in sweet and umami identification occurring already 1 month postsurgery (identification scores for umami it at T2 decreased when compared to T1, but were still higher than before surgery). The lack of a nonsurgical control group that was also evaluated three consecutive times is a study limitation, as such data would have provided an unconfutable proof to exclude learning effects, which could potentially contribute to the observed increases in postsurgery taste sensitivity, as well as to those of the smell identification. However, since subjects were evaluated at several weeks (to months) postsurgery (i.e., from their first sensory test), we expect learning effects to be of insignificant clinical relevance. Moreover, the value of the total taste score that we measured after six months of surgery approached values that are close to those determined in healthy normal-weight subjects [46,92], thus suggesting that an effect of learning may be unlikely. However, this cannot be persuasively excluded and it is certainly interesting that patients undergoing surgery return to normal in some subtle yet very important parameters. Although there are controversial data, some studies indicate that an increase in taste sensitivity may associate with a decrease in preferences of related foods [32,33,34,35,36,37,138,139,140,141]. Therefore, the increased ability to identify sweet taste that we found after bariatric surgery may contribute towards a reduced intake of high-calorie foods contributing to the success of the intervention. On the other hand, since umami taste is related to appetitive responses to protein [142], the increase in identification scores for umami that we found after surgery could explain the reduction in preferences for protein-rich food that are reported by subjects after surgery, which drastically reduce the consumption of this kind of food [84]. Our results also showed no effect of bariatric surgery on identification of quinine bitterness. This is not surprising given the liking that these patients show a for healthy dietary pattern is not associated with quinine bitterness but is mostly driven by lower sweet and refined carbohydrate liking [129]. Few studies are available on the relation between bariatric surgery and salt perception with inconsistent results [85,89]. The lack of changes in saltiness that we found after bariatric surgery is consistent with results showing bariatric surgery does not alter the salt detection threshold [85,143] or the hedonic responses evoked by cream soups differing in salt concentrations [143].

It is worth highlighting the indirect association that we found between the *OBPIIa* (A/G) locus and the variations in the overall taste sensitivity or sensitivity to sweet and sour tastes after surgery. Olfactory performance assessments and bioinformatics data suggested that the presence of the mutation in this locus decreased the expression of OBPIIa protein in the olfactory epithelium [71]. Our results showed that only the carriers of the G allele showed an increase in the overall taste sensitivity and sweet and sour tastes after surgery. This observation leads us to speculate that the increase in taste sensitivity after surgery might be more effective in subjects who have a minor expression of OBPIIa protein. Future studies will have to explore this hypothesis. Contrarily, the changes in the overall taste sensitivity or sensitivity to single taste qualities after surgery was not dependent on the PROP taster status of subjects. However, we observed a main effect of the PROP taster status on the total taste score and on bitter score, such that taster subjects (super-tasters and medium tasters) had higher scores than non-tasters at each time point. These findings fit with data showing a greater general taste sensitivity in tasters than non-tasters [26,27,28,29,30,31,32,144,145].

Our results also showed that the subjects in this current study (all with obesity) had a higher fat threshold (about 3-fold) with respect to that determined in normal-weight subjects in our previous study [31]. In addition, we found a significant increase in fat sensitivity after surgery determining oleic acid threshold values decreased with time after surgery, and six months postsurgery became significant lower than before surgery and similar to those observed in normal-weight subjects (0.22 µL) [31]. These results may explain the drastic reduction in preferences for high-fat foods that have been shown after bariatric surgery. Contrary to expectation, the positive effect of bariatric surgery on fatty acid taste was independent of *CD36* locus. All genotype groups had the same trend—i.e., that oleic acid thresholds decreased after surgery, though the effect seems more evident in subjects homozygous for the non-taster variant (AA). This could be due to the fact that the fat taste sensitivity is an individual feature complex and other factors can be involved, especially in subjects with overweight or obesity. It is known that habitual diet and BMI could influence taste sensitivity [21,61,146]. In addition, it is known that the CD36 expression in papillae decreased in high-fat diet-induced obese rats [147] and the exposure to, or restriction from, dietary fat can modulate taste sensitivity [146].

Consistent with previous studies [92,94,95], our results showed that the odor identification increased after bariatric surgery indicating an improvement of olfactory function of these patients. The increased olfactory sensitivity associated with weight loss and improvement of body composition that we found after surgery is consistent with data that showed that an increased ability of the olfactory bulb neurons (via modulation of Kv1.3 channel) contributes to the improvement of metabolic function and energy consumption [148]. Consequently, since an olfactory role has been shown in the modulation of PROP and oleic acid sensitivity [68], the increase in PROP and oleic acid sensitivity that we find not to be related to variants of the specific receptor (TAS2R38 or CD36, respectively), might be mostly explained by increased smell sensitivity or by consequent improvement of metabolic function. Surprisingly, we did not find a specific effect of *OBPIIa* locus on changes of odor identification after surgery given that similar trends for all genotypes were found.

Finally, our results showed that subjects with obesity, especially tasters, had high scores in cognitive restraint factor before surgery. In our study, scores for the restrained factor were much higher (≥10, median value) than those previously reported in the literature [103,104]. We hypothesize that these high scores are due to the educative training these subjects received before surgery, which were designed to re-establish a correct eating habit. Furthermore, a conscious control of eating was a fundamental inclusion criterion for being qualified for the surgery, since it has already been associated to a long-term weight loss success [149,150,151,152]. Consistently with previous works [108,109,110,111,112,129], our results also showed an increase in cognitive restraint and a decrease in disinhibition and perceived hunger after surgery. These findings seem to indicate that bariatric surgery can have a positive effect on cognitive control of eating behavior turn to contribute to the success of the intervention. In fact, a neuroimaging study indicated that bariatric surgery-related decreases in preference for unhealthy foods and increases in preference for healthy foods arise from changes in the network of frontoparietal control, which involve cognitive control of food sensations, while it failed to find involvement of reward-related brain regions [153]. The increase in restraint after surgery was associated with the PROP phenotype of subjects. Non-tasters and medium tasters showed increased values after surgery, while no significant changes in super-tasters were found. These observations lead us to speculate that non-tasters and medium tasters, compared to super-tasters, might need a higher restraint to be able to control their irregular eating behavior dictated by their lower taste sensitivity. Further investigation is needed to clarify this issue.

## 5. Conclusions

Our findings show improved olfactory and gustatory functions after bariatric surgery. Increases in sweet, umami and fat perception, together with increased cognitive restraint and decreased disinhibition and hunger, may contribute to the decrease in the preference and consumption of foods high in calories, sugar, fat, and protein reported after surgery [12,78,80,81,82,83,84,85,86,87,88], therefore contributing to the loss of weight and improvement of body composition in patients following bariatric surgery. In addition, our results suggest that genetic factors, such as *OBPIIa* gene polymorphisms and the heritable variation in PROP taste sensitivity, can play important roles in the bariatric surgery-induced changes of taste function and cognitive control of eating behavior.

## Figures and Tables

**Table 1:** Demographic, clinical and genetic features of subjects.

Clinical Features			
Surgery (*n*)	SG (21)	RYGB (30)	
Age (y)	42.28 ± 2.33	46.83 ± 2.08	
Female (*n*)	14	22	
Male *(n)*	7	8	
**Genetic Features**			
*TAS2R38 n* (%)	PAV/PAV 10 (21.28)	PAV/AVI 19 (40.42)	AVI/AV I18 (38.30)
*CD36 n* (%)	GG 14 (27.45)	GA 23 (45.10)	AA 14 (27.45)
*OBPIIa n* (%)	AA 15 (29.41)	AG 12 (23.53)	GG 24 (47.06)

**Table 2:** Genotype distribution and haplotype frequency of *TAS2R38* single nucleotide polymorphisms (SNPs) according to PROP taster status before (T0), one month (T1) and six months (T2) after bariatric surgery.

	PROP Status						*p*-Value
	Super-Taster		Medium Taster		Non-Taster		
*TAS2R38*	*n*	%	*n*	%	*n*	%	
**T0**							
*Genotype*							
PAV/PAV	1	25.0	9	32.1	0	0.0	0.00098
PAV/AVI	2	50.0	13	46.4	4	26.7	
AVI/AVI	1	25.0	6	21.4	11	73.3	
*Haplotype*							
PAV	4	50.0	31	55.4	4	13.3	0.0003
AVI	4	50.0	25	44.6	26	86.7	
**T1**							
*Genotype*							
PAV/PAV	4	26.7	6	23.1	0	0.0	0.0019
PAV/AVI	8	53.3	11	42.3	0	0.0	
AVI/AVI	3	20.0	9	34.6	6	100.0	
*Haplotype*							
PAV	16	53.3	23	44.2	0	0.0	0.0026
AVI	14	46.7	29	55.8	12	100.0	
**T2**							
*Genotype*							
PAV/PAV	4	40.0	6	21.4	0	0.0	0.0004
PAV/AVI	6	60.0	12	42.9	1	11.1	
AVI/AVI	0	0.0	10	35.7	8	88.9	
*Haplotype*							
PAV	14	70.0	24	42.9	1	5.6	0.000004
AVI	6	30.0	32	57.1	17	94.4	

*p*-value in derived from Fisher’s method (Genepop software version 4.2) (*n* = 47) (participants with rare haplotype are not included in the analysis).

## Data Availability

The data presented in this study are available on request from the corresponding author. The data are not publicly available in accordance with consent provided by participants on the use of confidential data.

## Supplementary Materials

The following are available online at https://www.mdpi.com/2072-6643/13/1/250/s1: Table S1: Anthropometric parameters determined before (T0), one month (T1) and six months (T2) after bariatric surgery; Figure S1: Bariatric surgery-induced effects on scores of sweet, sour, salty, bitter, and umami taste perceptions according to the *rs2590498* polymorphism of *OBPIIa* gene or PROP taster status.
