# Supplementary material for: Changes of Taste, Smell and Eating Behavior in Patients Undergoing Bariatric Surgery: Associations with PROP Phenotypes and Polymorphisms in the Odorant-Binding Protein OBPIIa and CD36 Receptor Genes
Source: Nutrients. 2021 Jan 16;13(1):250. doi: 10.3390/nu13010250 (PMC7830302; doi:10.3390/nu13010250)
Supplement: Supplementary file 1 [file nutrients-13-00250-s001.pdf]

Supplemental Material:

Bariatric surgery-induced effects on anthropometric parameters

Table S1 shows mean values (±SEM) of the anthropometric parameters defining the body composition determined in the participants before (T0), one month (T1) and six months (T2) after bariatric surgery. Repeated-measures ANOVA showed that all parameters significantly varied with time (BW: $F_{(1.34,66.84)}$ = 331.70, $p < 0.00001$; BMI, $F_{(1.42,71.09)}$ = 420.28, $p < 0.00001$; % TWL: $F_{(1,50)}$ = 528.15, $p < 0.00001$; % EWL: $F_{(1,50)}$ = 513.13, $p < 0.00001$; Neck: $F_{(1.13,55.18)}$ = 13.042, $p = 0.00041$; Waist: $F_{(1.46,71.73)}$ = 121.95, $p < 0.00001$; Hip: $F_{(2,100)}$ = 231.57, $p < 0.00001$; WHR: $F_{(1.50,73.45)}$ = 10.483, $p = 0.0004$; FFM: $F_{(1.67,80.11)}$ = 111.78, $p < 0.00001$; FM: $F_{(1.26,60.49)}$ = 79.599, $p < 0.00001$; TBW: $F_{(1.09,52.18)}$ = 3.216, $p = 0.0050$; % FFM: $F_{(1.62,78.01)}$ = 159.12, $p < 0.00001$; % FM: $F_{(1.55,74.68)}$ = 216.12, $p < 0.00001$; % TBW: $F_{(2,100)}$ = 3.291, $p = 0.041$). Post hoc comparisons showed that values of BW, BMI, circumferences of waist, hip, and neck, FFM, FM, TBW and % FM decreased from T0 toT1, and decreased even further from T1 to T2, when patients lost 28% of total weight and 68% of excess of weight. The opposite was true for %FFM, which increased from T0 to T1 and increased even further from T1 to T2. There were no significant differences related to type of surgery ($p > 0.05$) (data not shown).

Table S1. Anthropometric parameters determined before (T0), one month (T1) and six months (T2) after bariatric surgery.

	T0			T1			T2			P^a	P^b
BW (kg)	115.42	±	3.67	99.85*	±	3.02	82.40*#	±	2.62	<0.0000	< 0.0000
BMI (kg/m^2)	43.20	±	0.79	37.35*	±	0.57	30.78*#	±	0.51	<0.0000	< 0.0000
% TWL				13.11	±	0.71	28.20#	±	0.91	<0.0000	< 0.0000
% EWL				29.76	±	1.41	68.55#	±	1.76	<0.0000	< 0.0000
Neck (cm)	40.96	±	0.60	37.83*	±	0.54	36.46*	±	1.13	0.00041	≤ 0.0007
Waist (cm)	119.95	±	2.37	107.05*	±	1.98	90.81*#	±	2.10	<0.0000	< 0.0000
Hip (cm)	132.19	±	1.78	120.68*	±	1.65	107.80*#	±	1.26	<0.0000	< 0.0000
WHR	0.91	±	0.01	0.89	±	0.01	0.84*#	±	0.01	0.0004	≤ 0.0015
FFM (kg)	61.66	±	2.17	56.63*	±	2.00	52.43*#	±	2.02	<0.0000	< 0.0000
FM (kg)	54.73	±	2.11	44.11*	±	1.66	31.81*#	±	1.98	<0.0000	< 0.0000
TBW (l)	46.50	±	1.87	42.51*	±	1.67	40.76*	±	1.98	0.0050	≤ 0.0073
% FFM	52.99	±	0.76	55.99*	±	0.78	62.92*#	±	0.78	<0.0000	< 0.0000
% FM	47.17	±	0.75	44.06*	±	0.78	36.64*#	±	0.74	<0.0000	< 0.0000
% TBW	75.78	±	0.56	75.68	±	0.54	76.45*#	±	0.57	0.041	≤ 0.043

Values (means ± SE). Parameters defining body composition: body weight (BW); body mass index (BMI); total weight loss (TWL); excess weight loss (EWL); waist-hip-ratio (WHR); fat-free mass (FFM); fat mass (FM); total body water (TBW). (n = 51). P^a-value derived from repeated measures of ANOVA. P^b-value derived from Fisher LSD Post Hoc test; * indicate a significant difference with respect to T0; # indicate a significant difference with respect to T1.

Bariatric surgery-induced effects on scores of sweet, sour, salty, bitter and umami taste perception according to the rs2590498 polymorphism of OBPIIa gene or PROP taster status

The mean values (±SEM) of the score for sweet, sour, salty, bitter and umami taste perception determined before (T0), one month (T1) and six months (T2) after bariatric surgery are shown according to the *rs2590498* polymorphism of *OBPIIa* gene or PROP taster status in Figure S1. Repeated measures of ANOVA showed that the changes in the sweet and sour scores across time were associated with *OBPIIa* locus (sweet: $F_{(3.66,87.94)}$ = 3.169; $p = 0.020$; sour: $F_{(4,96)}$ = 4.107; $p = 0.0041$) (Figure S1A). The sweet scores determined at T1 and T2 in the participants who carried the GG genotypes were higher than that determined at T0 ($p \leq 0.027$, Fisher's test LSD), while no differences in participants who carried the AA or AG genotype

were found ($p > 0.05$). The sour score determined at T2 in the participants who carried AG and GG genotypes was higher than those determined at T0 ($p \leq 0.0038$, Fisher's test LSD), while no differences in participants who carried AA genotype were found ($p > 0.05$). There were no significant interactions between *OBPIIa* locus and changes in taste scores for salty, bitter or umami across time ($p > 0.05$).

Differently, the changes relative to each taste quality observed within time factor (T0, T1 and T2) did not associate with PROP taster status of participants. However, a significant main effect of the PROP taster status on bitter score were found ($F_{(2,148)} = 12.893$; $p = 0.00001$), such that super-tasters and medium tasters had higher scores than non-tasters ($p \leq 0.027$, Fisher's test LSD) (Figure S1B). No other difference related to PROP taster status was found ($p > 0.05$).

There were no significant differences related to gender or type of bariatric surgery ($p > 0.05$; data not shown).

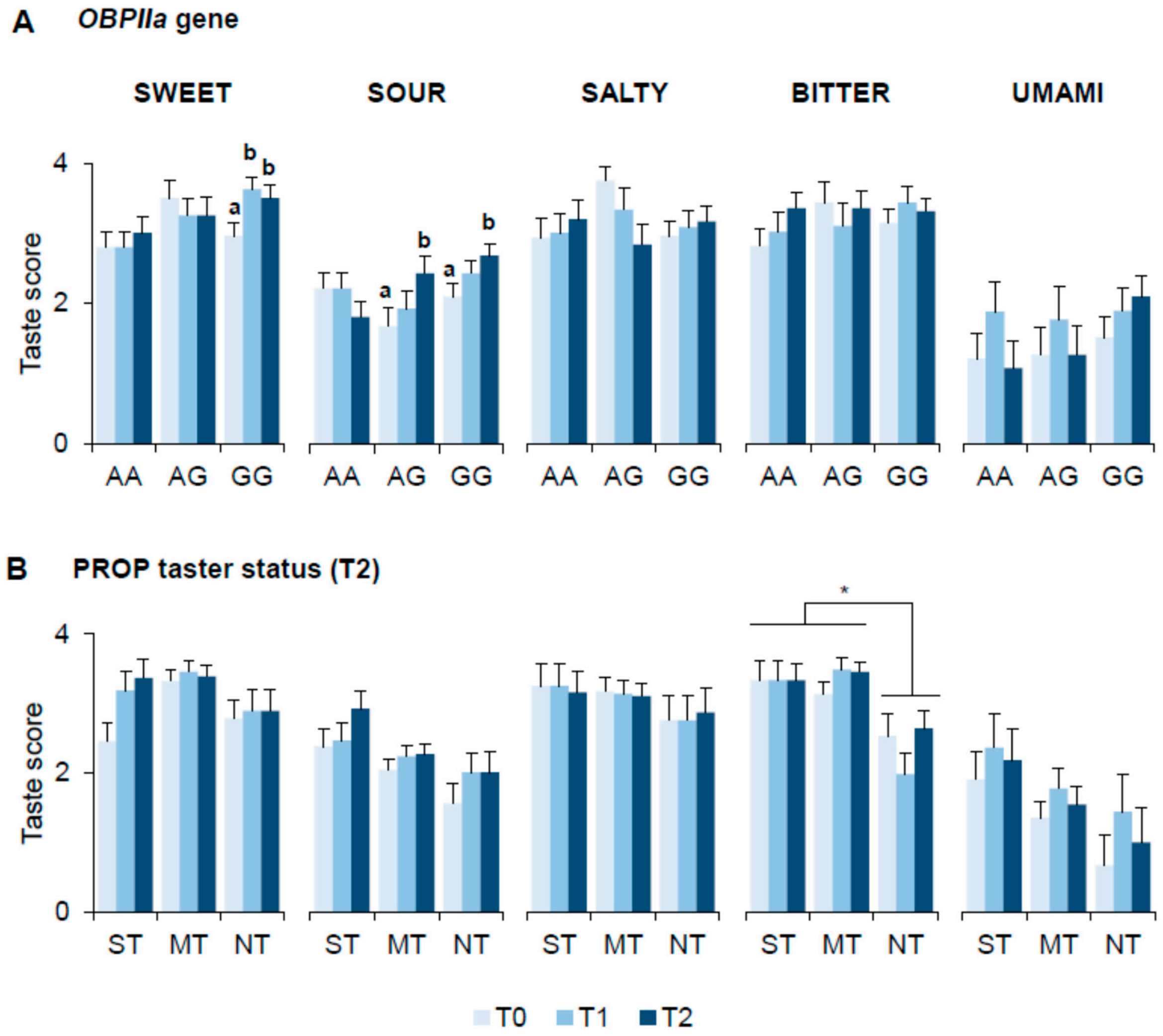

Figure S1. Taste perception scores relative to sweet, sour, salty, bitter and umami determined before (T0), one month (T1) and six months (T2) after bariatric surgery ($n = 51$). Means (±SE) values are shown according to the *rs2590498* polymorphism of *OBPIIa* gene (genotypes AA: $n = 15$; genotypes AG: $n = 12$; genotypes GG: $n = 24$) (A) or PROP taster status determined at T2 (super-tasters: $n = 11$; medium tasters: $n = 31$; non-tasters: $n = 9$) (B). Different letters indicate a significant difference ($p \leq 0.048$, Fisher's test LSD subsequent repeated measures ANOVA). * indicate a significant difference between values of tasters and non-tasters ($p \leq 0.027$ Fisher's test LSD subsequent repeated measures ANOVA).
